# European brain research area: The strategic level

**DOI:** 10.1192/j.eurpsy.2021.171

**Published:** 2021-08-13

**Authors:** M. Di Luca, E. De Witte, K. Aarts, S. Kramer, D. Iannone, F. Destrebecq

**Affiliations:** Eu-projects, European Brain Council, Bruxelles, Belgium

**Keywords:** Brain research, Coordination, Shared European Brain Research Agenda, Funding

## Abstract

Brain research in Europe is a rapidly evolving field, and increasingly at the forefront of science. Although considerable amounts of knowledge and innovative approaches have been generated, the translation into new health interventions is hindered by excessive fragmentation. Effective and efficient collaboration and cooperation among the various initiatives are often identified as a key success factor to achieve brain research full impact. EBRA fully responds to these needs by bringing together the various stakeholders and major brain research initiatives, at European level and beyond. EBRA creates the conditions for real and effective cross fertilisation, dialogue, building consensus and exploiting research potential. At the strategic level, EBRA acts by fostering alignment and better coordination of research strategies across European and global brain initiatives. Therefore, an overview of the scale and scope of brain research activities funded in the EU framework programme and the funding initiatives of JPND, NEURON and HBP has been created. The results of the mapping exercise then underpinned the development of a Shared European Brain Research Agenda (SEBRA). The SEBRA focuses on research opportunities and research gaps to be addressed in the field, and priorities for action in the short- and long-term. It integrates pre-existing documents as well as expert (i.e., researchers, neurologists/psychiatrists, patient representatives) input that has been collected through surveys and in a dedicated expert workshop. The SEBRA will be used to provide recommendations on future areas for excellent, innovative, and translational research comprising those for maximized cooperation, reduced overlap, and fragmentation.

**Disclosure:**

No significant relationships.

